# Microfluidic Device for the Identification of Biological Sex by Analysis of Latent Fingermark Deposits

**DOI:** 10.3390/mi12040442

**Published:** 2021-04-15

**Authors:** Jamila S. Marshall, Madelyn L. Sita, James P. Landers

**Affiliations:** 1 Department of Chemistry, University of Virginia, Charlottesville, VA 22903, USA; jsm2dg@virginia.edu (J.S.M.); ms4sc@virginia.edu (M.L.S.); 2 Department of Electrical and Computer Engineering, University of Virginia, Charlottesville, VA 22903, USA; 3 Departments of Mechanical Engineering and Pathology, University of Virginia, Charlottesville, VA 22903, USA

**Keywords:** bioanalysis, centrifugal, colorimetric analysis, fingermarks, latent fingerprints, microfluidic, forensics

## Abstract

To date, most research regarding amino acid detection and quantification in fingermarks relies on spectrometric methods. Herein, the Sakaguchi colorimetric test was adapted to a rotationally-driven microfluidic platform and used to detect and quantify arginine in fingermarks deposited by male and female donors. A red color indicates the presence of arginine in a given sample following the reaction, and the intensity of this color is linearly proportional to the concentration. Objective detection and quantification of arginine were accomplished using image analysis software (freeware) based on this colorimetric result. The mean concentrations obtained in a blind study were 96.4 ± 5.1 µM for samples from female donors and 55.3 ± 5.3 µM for samples from males. These were not statistically different from the literature values of 94.8 µM ± 12.9 µM for females (*p* = 0.908) and 54.0 ± 12.6 µM for males (*p* = 0.914), respectively (± SEM in all cases). Conversely, the experimental means from males and female samples were statistically different from each other (*p* < 0.001). Objective differentiation between male and female fingermark deposits was achieved in a blind study with 93% accuracy. Additionally, the method was compatible both with samples lifted from common surfaces and with magnetically-powdered samples.

## 1. Introduction

Fingerprint analysis is an indispensable tool used in forensic and criminal investigations. Despite the emergence of state-of-the-art DNA analysis technologies, it was reported that fingerprints outperform these and other comparable identification systems in the discernment of dangerous offenders [1]. Another pertinent example of the indispensability of these analyses is the discovery that identical twins have discernible fingerprints despite possessing indistinguishable DNA genetic profiles [2]. Such a distinction supports the premise that fingerprints serve as an invaluable investigative tool. Although the biometric analysis of the physical fingerprint pattern is quite prevalent, there has been significantly less research regarding the potential use of fingerprints as biological samples comparable to other body fluids such as blood or saliva. Development in fingerprint analysis has primarily been confined to visual or digital comparison and matching of prints for approximately the past 110 years. Conversely, research regarding the viability of fingermarks as biological samples has only become prevalent in the last decade [3,4].

When considering the use of fingerprints in this capacity, it is essential to note the distinction between the common term “fingerprint” and a term often used in the forensic community in such cases, “fingermark”. Fingerprints refer to the unique, physical pattern emplaced following contact between the finger’s papillary skin and another surface. Fingermarks are the “biological material transferred from the surface of the skin to another surface on contact” [5,6]. Latent fingermarks and fingerprints refer to those which are poorly visible or undetectable to the unaided eye but can be developed for analysis. This inherent difference can serve to address the deficiencies of current fingerprint analysis techniques [7].

Latent fingermarks are primarily comprised of sweat secretions from the sebaceous and eccrine glands and incorporate many metabolites arising from hormone-regulated metabolic mechanisms [3]. These metabolites can then be regarded as biomarkers or indicators of other physical and physiological properties such as age, ethnicity, health status, and biological sex [3,8]. 

Traditionally, fingermark development has primarily been achieved using colored dyes or stains. The methods implemented typically require chemical development in a laboratory and are not used at a crime scene [9]. The treatment employed is usually dependent on the deposition surface. For porous samples like paper or cardboard, liquid chemical treatments such as 1,2-diazafluoren-9-one (DFO) development and the well-known ninhydrin are most often used [9,10]. The ninhydrin method has been used in federal, city, and state crime laboratories for almost 50 years. However, an inherent drawback of this reaction is that the end-product, diketohydrindylidene-diketohydrindamine (DYDA) or Ruhemann’s purple, may also be developed from other α-NH_2_ compounds such as ammonium salts. This cross-reactivity gives rise to the potential for interference [10]. In cases where fingermarks are deposited on smooth, non-porous surfaces, development is often achieved using cyanoacrylate (commonly known as superglue) vapor or vacuum-metal deposition [9]. These chemical techniques discern the latent fingermark but do not provide detailed biological information about its content. 

To date, most studies in this area of fingermark analysis have been conducted using spectrophotometric methods [3,5]. One such example is the successful development of chemical and enzymatic assays targeting amino acids present in fingermarks for use as biomarkers by Brunelle et al. [3,4,10,11]. Specifically, determination of the biological sex of the fingermark originator has been identified as a means of linking a specific analyte to one originator attribute. This differentiator was intended to serve as a basis for the eventual expansion of the protocol to include the identification of multiple characteristics via individual markers or analytes [4]. In this study, detection was achieved spectrophotometrically using UV–vis analysis. While there are many advantages inherent to spectrophotometric analysis as relates to precision and sophistication, colorimeters are comparatively less expensive and can be more compact, mobile, and simple to operate [12]. As such, a rapid, inexpensive, simple, and portable device of this type for analysis of the biological contents of fingermarks would be beneficial for use at crime scenes [4].

It is useful to note that although males are statistically more likely to be involved in criminal activities than women, research has persisted in this area for multiple reasons [13]. Firstly, it is believed that analysis of fingermark content can be instrumental in cases where there is no reference fingerprint in a database to which a crime scene fingerprint could be compared for individualization [14]. Additionally, analysis of deposits could provide biological information about the perpetrator in cases where there is distortion or deterioration of fingermarks [14]. 

Chemical and enzymatic assays conducted in previous experiments have shown that females have an overall higher concentration of amino acids in their sweat than males [3,4,15]. In most cases, the concentrations of amino acids in the fingermarks of female origin are approximately twofold those obtained from males. As such, amino acid concentration in fingermark material can indicate the biological sex of the source.

While enzymatic assays present certain advantages such as specificity and sensitivity, there are also disadvantages inherent to using such methods. Enzymes are less stable than many chemical components and can have a limited shelf-life. There is thus a requirement for carefully controlled storage conditions and more frequent replacement. Consequently, a specific and sensitive chemical assay is more widely applicable for detecting amino acids in fingermark content. One such chemical assay is the Sakaguchi Test for the detection of arginine. In this test, arginine, sodium hypobromite, and α-naphthol react under alkaline conditions to produce a red-brown complex [4,16]. The intensity of the color is directly indicative of the concentration of arginine present in the sample. This test is applicable in determining biological sex from fingermarks as the average concentrations of arginine in males’ and females’ sweat differ significantly. There is an average concentration of 54.0 ± 12.61 µM (SEM) in males, while in females, the average concentration in sweat is 94.8 ± 12.86 µM (SEM) [15]. Herein, SEM refers to the standard error of the mean and is indicative of the dispersion of sample means around the population mean, thereby accounting for sample size [17]. A definitive colorimetric result also allows for the analysis of results using open-source image analysis software, namely, the Fiji (Fiji Is Just ImageJ) distribution of ImageJ [18,19]. This protocol presents a lower-cost alternative to detection by UV–vis spectrophotometry. It is advantageous as the only requirements are the software (freeware) and a standard digital scanner. In concert with the scanner, the proposed microdevice measures the arginine content in fingermarks using non-enzymatic assays, thereby quickly and objectively determining unknown individuals’ biological sex. We note that (to our knowledge) this is the first microfluidic device capable of facilitating objective, cost-effective analysis of fingermark deposits via optimization of the Sakaguchi reaction. Additionally, this technology can be further adapted to allow for an integrated, automated, and portable analysis system. 

## 2. Materials and Methods

### 2.1. Image Analysis 

All images were analyzed using FIJI software. Following color development in each case, the detection domains were scanned using an Epson Perfection^®^ V600 Photo Scanner (Epson America, Inc., Plainfield, IN, USA) and saved as TIFF files for image processing and analysis. Target segments were further isolated using the color thresholding feature of Fiji [19,20]. The images were first analyzed using the RGB (red, green, blue) color space, and results were generated in standard units in the 0–255 range. Images were then converted using the red to magenta conversion plugin of the software and retested using the RGB color space. This step was followed by a conversion from the RGB to the HSB (hue, saturation, brightness) color space to analyze saturation. Results generated for saturation were reported as a percentage (0–100%). 

### 2.2. Proof of Concept—Sakaguchi Reaction and Modified Detection Method 

Standard solutions with concentrations of 20, 40, 60, 80, 100, and 120 µM of L-arginine (Fisher Scientific, Hampton NH, USA) were prepared by dissolution in Millipore water. Stock solutions of 1.25 M and 2.50 M sodium hydroxide (Sigma-Aldrich, St. Louis, MO, USA) and 1.5 mM α-naphthol (Alfa Aesar, Haverhill, MA, USA) in 95% ethanol (*v/v*) (Sigma-Aldrich, St. Louis, MO, USA) were prepared. Additionally, a stock solution of sodium hypobromite was made by combining 32 µL of bromine (99+%, Acros Organics, Morris, NJ, USA) and 1.25 M sodium hydroxide to achieve a total volume of 5 mL. For each standard, 150 µL was added to a 1.5 mL centrifuge tube (USA Scientific, Inc., Ocala, FL, USA). This was followed by the addition of 30 µL each of 2.5 M sodium hydroxide and 1.5 mM α-naphthol solutions as outlined by Huynh et al. [3]. There was a standard addition of 3.0 µL of 2.5 mM L-arginine to each reaction to facilitate measurable color development. The reactants in each tube were mixed using a vortex mixer (Vornado™ Miniature Vortexer, Benchmark Scientific, Sayreville, NJ, USA) and incubated at 4 °C for 5 min. Following incubation, 5 µL of sodium hypobromite was added to the centrifuge tube, the reaction mixture was vortexed, and the tubes were scanned before image analysis was performed.

### 2.3. Device Fabrication

The device was created using the Print, Cut, and Laminate (PCL) fabrication method previously developed by the Landers’ lab [21]. The microfluidic device was first designed using Autodesk^®^ AutoCAD^®^ software, and the file was transferred to VersaLASER^®^VL3.50 software for the ablation of the design by the laser cutting instrument. Six layers comprised of black and transparent polyethylene (PET) (No Stripe Copier/Laser Transparency Film, Film Source Inc. (Tokyo Film Service Co. Ltd., Tokyo, Japan), heat-sealing adhesive (HSA) (Adhesives Research, Glen Rock, PA, USA), and polymethylmethacrylate (PMMA) (McMaster Carr, Santa Fe Springs, CA, USA). After programmed cutting of the layers, any extraneous obstructions remaining in the vents, channels, and inlets were removed using tweezers. The layers were then combined using a custom alignment tool and laminated at 180 °C by passing the aligned structure through an office laminator (Akiles UltraLam 250 B). 

### 2.4. Fingermark Deposition and Arginine Extraction—Pilot Tests

Fingermarks deposits were collected from volunteers who were assigned a reference code to protect their identities. Still, the biological sexes of the donors were known by the primary researcher to confirm proof of concept. Volunteers were asked to individually place their fingertips onto a designated area on PET transparency film (No Stripe Copier/Laser transparency Film, Film Source Inc.) and directed to maintain contact for approximately 5 s. Fingermark deposits at this stage were “natural” as there were no specific preparatory instructions given to volunteers before placement of deposits. The relevant segment of the transparency was then removed and placed in a 35 × 10 mm sterile, polystyrene Petri dish (Falcon^®^ Disposable Petri Dishes, Corning, Corning, NY, USA) and stored at ambient temperatures (~24 °C) for ≥ 24 h.

After storage, 120 µL of 0.01 M HCl (Fisher Scientific, Waltham, MA, USA) stock solution was placed directly onto the fingermark. The cover was replaced, and the dish was heated at 40 °C for 20 min using a hotplate/stirrer (VWR 4x4 Ceramic Hotplate/Stirrer 120 V). The sample solution was then transferred to the microfluidic device by pipetting 100 µL of this liquid from the transparency film surface. The Sakaguchi reaction was then initiated using 22 µL each of 2.5 M sodium hydroxide and 1.5 mM α-naphthol [3,16]. There was a standard addition of 3.0 µL of 2.5 mM L-arginine to each reaction for measurable color development. The contents of the mixing chamber were mixed using a custom-built stepper spin system (Sanyo Denki SANMOTION Stepper Motor, Parallax Propeller microcontroller) for 30 cycles at 1500 rpm. Mixing was facilitated by quick, repeated lateral rotations of the disc clockwise and anticlockwise enabled by the motor. The reaction mixture (on disc) was then incubated at 4 °C for 5 min. Following incubation, the laser valve was breached via laser actuation using a Power and Time Adjustable Manual Laser (PTAML) (Thorlabs) (500 mW, 0.5 s). Sodium hypobromite (4 µL) was added to the microfluidic device’s detection window to initiate color development. The device was then rotated using a custom spin system (E-flite Park 450 Spin System, Parallax Propeller microcontroller) at 2750 rpm for 15 s to allow for the metered transferal of 140 µL to the detection domain and mixing with sodium hypobromite. The detection window was then scanned, and the images were analyzed.

### 2.5. Lifted Deposits

The substrates chosen for deposition of fingermarks were a standard laminate benchtop and a stainless-steel light switch. The intended location of deposition was first sanitized using a 10% bleach solution. The area was then wiped with 90% ethanol and allowed to dry naturally. Donors deposited fingermarks on the relevant substrate, and a section of PET transparency film (~9 cm^2^) was placed directly onto the deposits, with care taken to avoid sliding or smudging. As with the pilot tests, there were no preparatory instructions given to donors before the deposition of fingermarks. When the PET film was securely in place, pressure was carefully applied back and forth on the film’s upper surface for 5–7 s to allow for the transferal of deposits. The PET film was carefully lifted from the surface using precision tweezers, inverted, and placed in a 35 mm borosilicate Petri dish. This Petri dish was then stored at ambient temperature for ≥ 24 h before arginine extraction. Samples from matching donors were deposited directly onto PET film and used as controls.

### 2.6. Magnetically-Powdered Deposits

Fingermarks deposited onto PET film were dusted using magnetic, bichromatic fingermark powder (Lynn Peavey Company, Lenexa, KS, USA). The excess powder was removed using a magnetic brush, and the samples were stored for ≥ 24 h at ambient temperature before arginine extraction. Following acid hydrolysis, the hydrolysate was transferred from the surface of the deposit to a centrifuge tube. A strong magnet was placed under the tube to promote sedimentation of residual magnetic particles. A volume of 100 µL of the hydrolysate was then transferred to the microdevice to undergo the Sakaguchi reaction. Image and data analysis were subsequently executed. Duplicate samples from donors were deposited directly onto PET film and used as controls. 

### 2.7. Blind Study 

Volunteers were solicited from within the research group for the acquisition of fingermark deposits. There was no assigned ratio of biological sexes or prescribed age range. Volunteers were first asked to thoroughly wash hands using a generic antibacterial soap, which was provided. Volunteers were then asked to place their hands in provided standard nitrile, unpowdered gloves. A designated colleague was present to ensure that there was no contact with extraneous surfaces such as stationary, taps, and the outer surfaces of gloves. Subjects then placed their fingers firmly in designated slots on PET for 5 s each. The participants’ biological sexes were withheld from the researcher conducting experiments until presumptive IDs of sample donors had been established.

## 3. Results and Discussion

### 3.1. Color Space Selection

**Figure 1:**
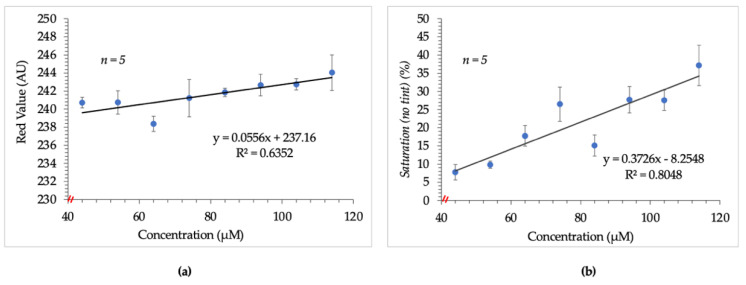
Results obtained from initial image analysis techniques. (**a**) shows the poor linear relationship between arginine concentration and average red value. (**b**) shows the slightly improved but non-ideal relationship observed between saturation and arginine concentration. Images were tinted prior to saturation measurement thereafter to address this issue.

Experiments were initially conducted in-tube to determine the feasibility of using the Epson V600 as the detector. Digital images obtained from the Epson V600 were to be analyzed using the FIJI image processing package [18,19]. The images were first examined based on the average “red value” of the RGB color space for each set of standards. It was presumed that since the resultant complex of the Sakaguchi reaction was red, the average red value from RGB would be proportional to the arginine concentration. Figure 1a depicts the poor linearity observed when increasing average red value was correlated to increasing concentration. This outcome suggested the potential for correlation but disqualified independent use of this parameter as a viable indicator. 

As such, the data set was analyzed using the saturation parameter of the HSB color space. Following this change, a slightly improved but non-ideal correlation between increasing arginine concentration and saturation values was observed. This result is shown in Figure 1b. 

**Figure 2:**
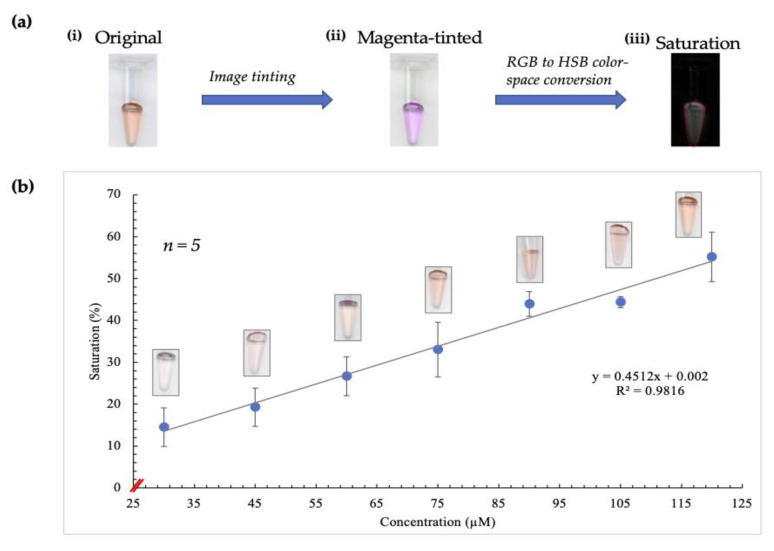
Illustration of results obtained using image-tinting techniques. (**a**) (**i**) shows a scanned image of a tube containing the complex formed after the Sakaguchi reaction was performed on a standard sample. The intensity of red color correlates directly to increasing arginine concentration. (**ii**) shows the image following red to magenta conversion in Fiji. Saturation (image shown in (**iii**)) was obtained using RGB (red, blue, green) to HSB (hue, saturation, brightness) color-space conversion, resulting in the observation of a more linear relationship between saturation and concentration. (**b**) shows the curve obtained when concentration was correlated with saturation values following image-tinting. The corresponding images obtained for each concentration are also depicted on this curve.

Given the improvements observed due to employing saturation measurement, the next step was to apply pre-existing image-tinting techniques previously developed in our lab to determine whether these were beneficial in this case [22]. Consequently, the red to magenta FIJI plugin was used before analyzing the average saturation values, as illustrated in Figure 2a.

In this conversion, the blue channel of the RGB color space is excluded and replaced with a copy of the red channel. Additionally, occurrences of red in a red/green image are converted to magenta, thereby creating a magenta/green merge [23]. This plugin was designed to accommodate individuals with red-green blindness, but in this case, it served to enhance the contribution of the red channel significantly. As a result, there was greater discernment of subtle differences in saturation, as shown by the plot obtained (Figure 2b).

A significantly improved correlation between concentration and saturation values was evident, and ergo, this combination of techniques was included in the image analysis stage of experiments thereafter.

### 3.2. Optimized Device Design 

**Figure 3:**
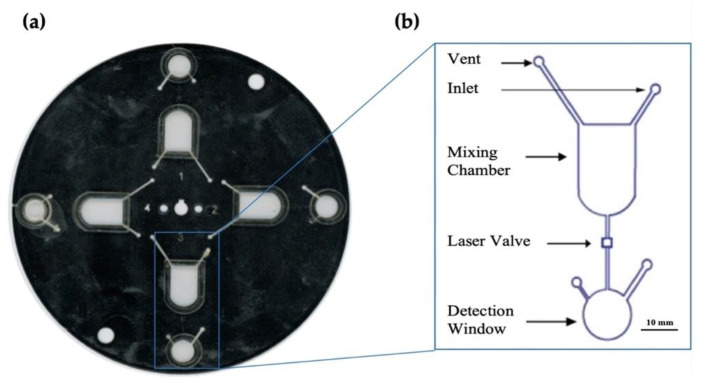
Optimized microfluidic disc. (**a**) shows a scanned image of the microfluidic disc designed for these experiments. (**b**) is a magnified schematic of the architecture of each microfluidic domain as designed using AutoCAD^®^ software. This disc was created using the Print, Cut, and Laminate technique previously developed by the Landers lab [21].

**Figure 4:**
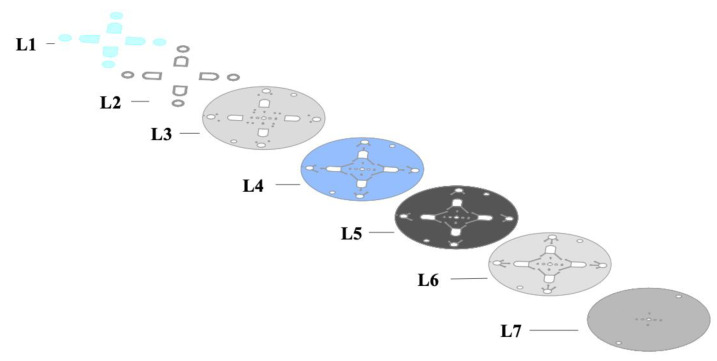
Schematic describing the individual layers of the microdevice used in these experiments. Layer **L1** is the PET layer used to seal microfluidic chambers. **L2** shows the PMMA segments used to increase the capacity of the fluidic chambers. **L3** and **L7** represent PET layers. **L4** and **L6** represent PET coated with HSA. **L5** represents a black PET layer that serves as a barrier between fluidic channels until laser actuation.

Following the determination of the most efficient image analysis protocol using in-tube experiments, the next step was creating and optimizing a disc that would best accommodate these parameters. The resulting optimized design of the device is shown in Figure 3. The disc features four discrete segments, each 90° to the neighboring section. The shape of the microdevice evolved from an initially rectangular architecture to a circular disc to maximize the benefits of centrifugal microfluidic mixing and facilitate multiple testing segments per disc. All iterations of the chip were five-layer devices with the addition of PMMA to increase the chamber depth and capacity and PET to cover the expanded chambers. The first uncoated PET layer defines the inlets, compartments, and vent holes of the device. The second and fourth layers demarcate the chambers as well as the channel barriers. The third toner-coated layer is a barrier between the second and fourth layers before the required fluid transferal between chambers. The fifth layer covers any exposed channels and chambers to create an enclosed system. Layers four and six are coated with a heat-sealing adhesive (HSA), which facilitates the binding of layers in the lamination process. These layers are illustrated in Figure 4.

**Figure 5:**
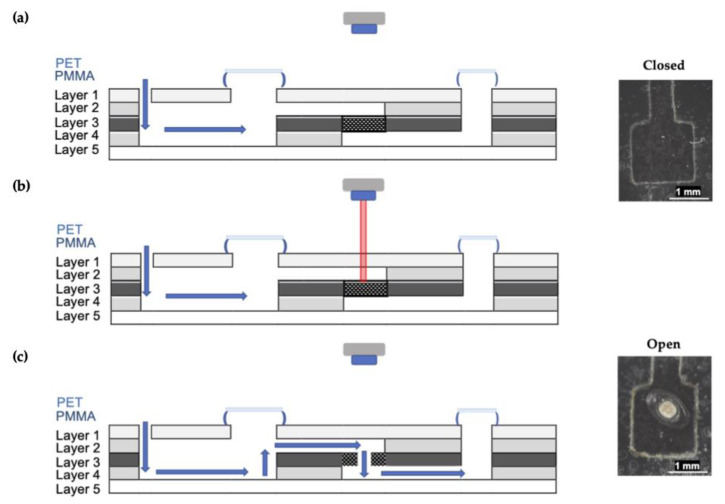
Schematic depicting a lateral view of microdevice architecture. (**a**) Reagents are introduced to the device via the inlet and held in the upper chamber by a closed laser valve. Mixing occurs via rapid rotations using the spin system. (**b**) The toner patch absorbs energy, and the PET layer is melted. (**c**) The solution can then be transferred from the first chamber to the second via the open laser valve.

The reagents (2.5 M NaOH, 1.5 mM α-naphthol in ethanol) and sample or standard solutions are introduced via inlet to the mixing chamber, and mixing was via the external, centrifugal microfluidic system. The barrier was breached by melting the valve using a custom laser, followed by color development in the detection window as previously described. The generation of centrifugal forces promotes transfer through the use of high-frequency, unidirectional rotations. Figure 5 details a cross-section of the microdevice and illustrates how valve ablation facilitates fluid transfer. 

### 3.3. Pilot Tests

**Figure 6:**
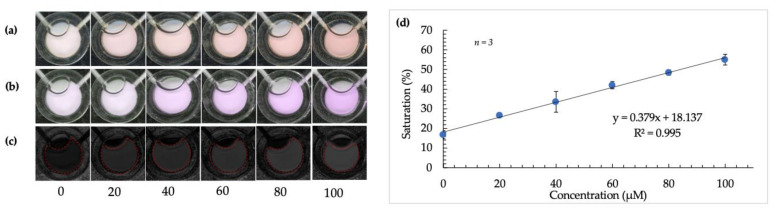
Calibration curve using optimized microdevice and image-analysis techniques. (**a**–**c**) show images of detection windows with increasing color intensity as arginine concentration increases (from left to right) before image tinting, following image tinting, and after RGB to HSB color-space conversion, respectively. (**d**) is a plot showing the robust and linear correlation between saturation value and increasing concentration of arginine in fingermark deposits using a microdevice.

After color space selection and determination of the detection method’s feasibility, the reaction was conducted using volunteer samples and the optimized microdevice. The calibration curve obtained using the microdevice and image analysis techniques is shown in Figure 6 [20]. A significantly better R squared value of 0.995 was obtained in this case.

Subsequently, pilot experiments were conducted using a small number of donor samples (n = 7 male and 7 female). These samples were obtained via the voluntary donation of fingermark deposits from individuals of both biological sexes within the research group. As previously stated, volunteers were assigned a reference code to protect their identities, and there was no predetermined ratio of biological sexes. There was also no enrichment of fingermarks at this stage: no touching or rubbing of the nose or forehead before deposition. Omitting fingermark enrichment was intended to facilitate objective investigation of the potential to detect and analyze arginine content in non-ideal but realistic conditions. As such, data obtained would more closely approximate “natural” sweat content in deposits.

Additionally, there was a consistent delta observed between mean thumb and “little” finger arginine concentrations compared to those obtained for the other fingers. As such, it was determined that it would be prudent to focus analyses on the index, middle, and ring fingers. This delta was attributed to the difference in skin surface area in contact with the PET surface for the largest and smallest fingers.

**Figure 7:**
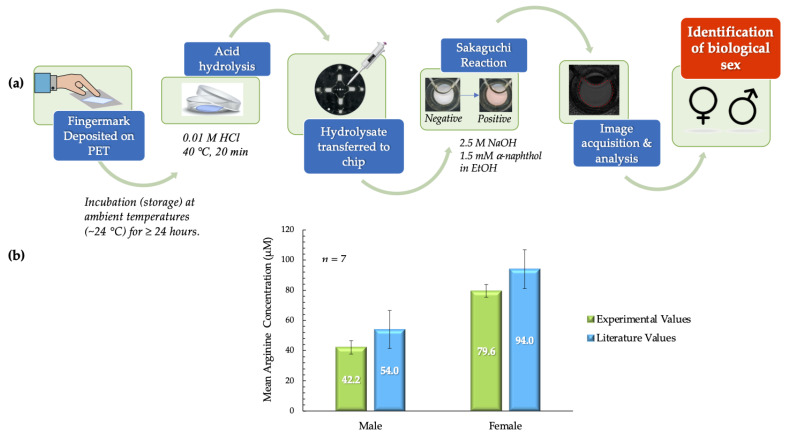
(**a**) Schematic outlining the process used to analyze arginine content in donor fingermark deposits and consequently determine biological sex. (**b**) Literature values for mean arginine concentration vs. experimental values in pilot tests. The mean arginine concentrations from male and female samples were significantly different (*p* < 0.001). Experimental values obtained for males and females were not statistically different from literature values (males - 42.2 ± 4.4 vs. 54.0 ± 12.6 µM (*p* = 0.367); females - 79.6 ± 4.3 µM vs. 94.8 µM ± 12.9 (*p* = 0.256) (experimental vs. literature, ± SEM in all cases) [15].

As illustrated in Figure 7a, the deposits were first placed on PET squares and incubated before further analysis. Post-incubation processes were completed in ~40 min with the inclusion of the acid hydrolysis step (20 min). In the acid hydrolysis step, the hydrophilic components of the fingermark deposits, such as amino acids, proteins, and salts, migrate to the hydrochloric acid solution. The hydrophobic PET surface retains the lipophilic components derived from sebaceous secretions. These phenomena enable the extraction of arginine from the fingermark deposit matrix. Following image and data analysis of all samples, the mean arginine concentrations obtained experimentally from males and females were compared to literature values and each other. These comparisons showed that there was no significant statistical difference. For males, the experimental mean obtained was 42.2 ± 4.4 µM, as compared to the theoretical value of 54.0 ± 12.6 µM [15]. For females, the mean arginine concentration obtained experimentally was 79.6 ± 4.3 µM, as compared to the literature value of 94.8 ± 12.9 µM [15]. The statistical similarities were confirmed for both data sets using the *t-*test. The *p* value obtained when experimental values for female samples were compared to literature values was 0.256. When this comparison was analyzed for male samples, a *p* value of 0.367 was obtained. Conversely, it was also determined that the mean arginine concentrations for male and female samples were significantly statistically different (*p* < 0.001). This information is summarized in Figure 7b.

### 3.4. Lifted Fingermark Deposits

In the pilot testing phase of these experiments, fingermarks were deposited on PET to evaluate the feasibility of the protocol and detection method. However, in a forensic investigation, fingermarks are found on other substrates, for example, wood, glass, doorknobs, laminate, or paper. Porosity, wettability, and other substrate characteristics play a

significant role in forecasting the longevity and viability of deposits [9]. It has been experimentally determined that up to three times more amino acid material is found in deposits on porous surfaces, e.g., paper and cardboard, than on non-porous substrates, e.g., glass and metal [9]. Eccrine sweat is also more readily absorbed than sebaceous secretions and is thus more efficiently transferred onto porous substrates. Sebaceous secretions, however, can remain on the substrate surface for up to several years following deposition [9]. It was therefore imperative to investigate this phenomenon under “real-world” conditions.

**Figure 8:**
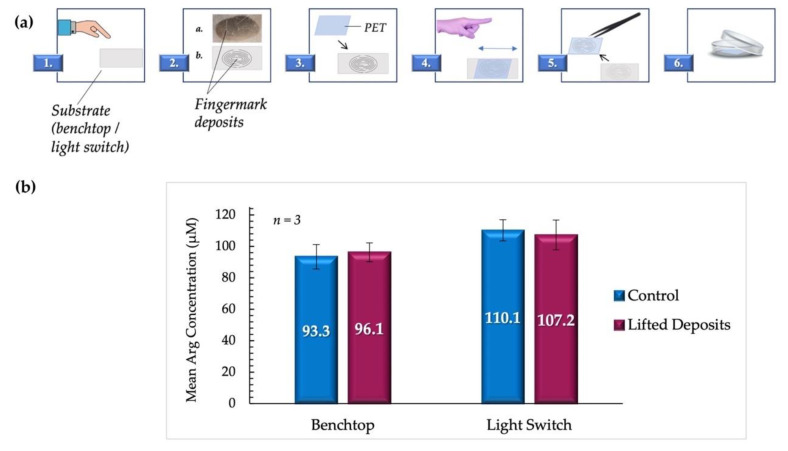
(**a**). Summary of the lifting process. **1.** The donor deposited fingermarks on the appropriate substrate. **2.** represents the fingermark deposits as they appear on the substrate, with **a.** showing a digital image of a fingermark deposit on a benchtop as obtained in the experiments, and **b.** showing a representative illustration. **3.** A section of PET transparency film (~9 cm^2^) was placed directly onto the deposits. **4.** Pressure was carefully applied back and forth on the upper surface of the film to allow for the transferal of deposits. **5.** PET film was lifted from the surface using precision tweezers, inverted, and **6.** placed in a 35 mm borosilicate Petri dish. (**b**) compares the mean arginine concentrations obtained from lifted vs. control fingermark deposits (female donors). Samples deposited directly on PET sheets were used as controls.

The surfaces selected in this case were a laminate benchtop and a stainless-steel light switch cover. These were located in the research lab and chosen due to their non-porosity. It was presumed that the low-porosity substrates would create a realistic but non-ideal set of conditions in addition to being commonly accessed locations. Consequently, the robustness of the proposed modifications would also be investigated. A schematic outlining the details of the lifting process is shown in Figure 8a. 

Given the paucity of arginine in fingermark deposits of male origin, it was probable that the inclusion of these samples could result in misleading results, whereby the scarcity of material would be incorrectly interpreted as an experimental design flaw or procedural failure. Consequently, proof-of-concept experiments were conducted using deposits from female donors only to reduce this uncertainty. This assertion was supported by the work carried out by Huynh et al., where the low signal from male fingermarks resulted in the use of deposits from females exclusively [3]. The results of this phase of tests are graphically displayed in Figure 8b.

It was observed that despite the variation of surfaces and the inclusion of lifting steps, there was no significant statistical difference between arginine concentrations in lifted fingermark deposits and those deposited directly onto PET as controls. For samples lifted from the benchtop, the mean arginine concentration obtained was 96.1 ± 6.7 µM, compared to controls with a mean of 93.3 ± 7.7 µM (*p* = 0.799). The mean arginine concentrations obtained for samples lifted from the light switch were 107.2 ± 9.5 µM and 110.1 ± 6.0 µM for lifted and control samples, respectively (*p* = 0.805). All samples are reported with standard error measurements. It was also determined that the concentration means of the lifted benchtop samples and the lifted light switch samples were not significantly different (*p* = 0.162). These findings support the potential for the application of the proposed protocol to crime scene forensic investigations.

### 3.5. Magnetically-Powdered Fingermark Deposits

Powders have been described as “probably the oldest and most common techniques for the enhancement of latent fingerprints” [9]. Fluorescent, magnetic, and carbon-based powders of various colors and compositions are routinely used to visualize latent prints at a crime scene. Given the inherently destructive nature of the proposed protocol, it was determined that the potential combination of techniques merited investigation. This combination would accommodate visualization of the print before experiments and chemical analysis, thereby allowing for the acquisition of both biometric and biochemical information.

**Figure 9:**
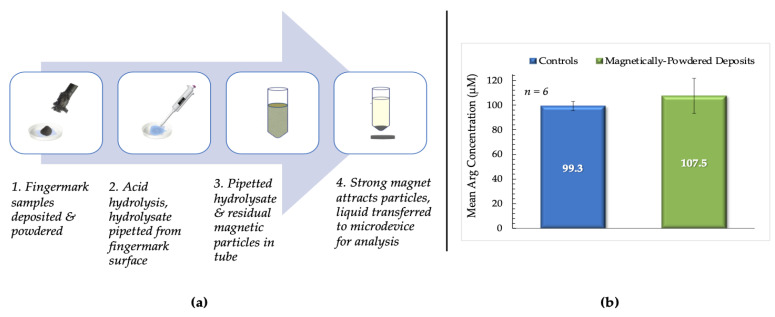
(**a**). Summary of the testing process for magnetically- powdered samples. **1.** Fingermarks were deposited on PET, placed in Petri dishes, and dusted using the magnetic powder and brush. Excess powder was removed from the deposits’ surface before incubation. **2.** Acid hydrolysis after incubation; hydrolysate then pipetted from the surface of the sample. **3.** Mixture of residual magnetic particles and hydrolysate transferred to a centrifuge tube. **4.** Strong magnet placed under the tube to promote sedimentation of the magnetic particles. Hydrolysate was then transferred to the microdevice and analyzed using the optimized protocol. (**b**) This bar chart compares mean arginine concentrations from magnetically-powdered vs. control fingermark deposits from female donors. Controls were unpowdered samples deposited on PET sheets.

Bichromatic magnetic powder was chosen due to its sensitivity and potential to be visualized on both light and dark surfaces. Additionally, excess powder can be removed using non-contact methods. This characteristic is beneficial as it reduces the chances of contamination or potential distortion of deposits. The process used for testing magnetically-powdered fingermark deposits is described in Figure 9a. 

It was determined that there was no significant difference between mean arginine concentrations obtained from magnetically-powdered fingermark deposits and unpowdered controls deposited on PET (*p* = 0.590), as shown in Figure 9b. Female donors were again used exclusively for these experiments to decrease the uncertainty regarding experimental conditions. The results obtained suggest the potential for combining visualization techniques with the proposed protocol and optimized microdevice to acquire more information about potential perpetrators at a crime scene. 

### 3.6. Blind Study

A blind study was conducted to analyze the robustness and applicability of the protocol conclusively. In the pilot testing phase of experiments, volunteers were assigned a reference code to protect their identities but were known to the researcher. For these experiments, however, the donors’ biological sexes were recorded by a colleague for post-experimental verification and withheld from the primary analyst to preclude experimental bias. Biological sex was then presumptively proposed based on the results obtained and subsequently determined to be correct or incorrect. 

Fingermark deposits were obtained from 16 unique participants. Of these, one set was rejected as there were no discernable prints or deposits. As with the pilot study, five impressions were taken from each donor, but the thumb and little finger deposits were excluded due to inconsistent and erratic concentration values resulting from differences in finger sizes. Consequently, three prints each from eleven females and four males were analyzed, and the results obtained from these experiments are summarized in Table 1. The biological sexes of all participants were correctly determined except for one case. As such, the accuracy of discernment between biological sexes was fourteen of fifteen (93%).

**Figure 10:**
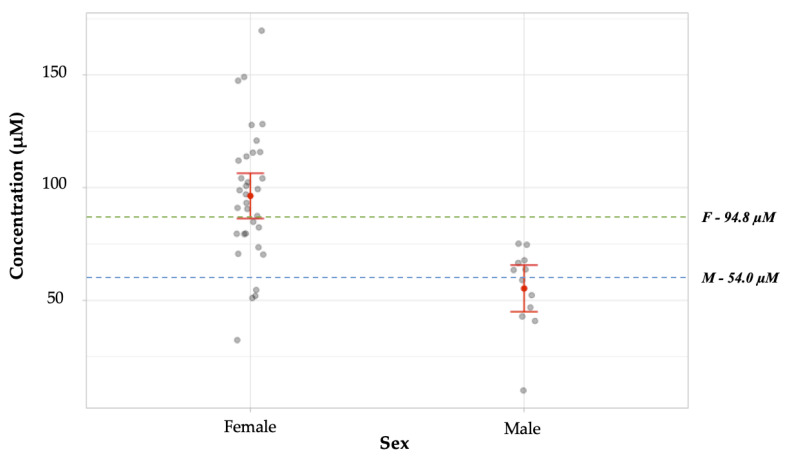
Comparison of mean arginine concentrations obtained from male vs. female individual samples in a blind study. These means were found to have a significant statistical difference (*p* < 0.001). For males (*n =* 12), the mean obtained was 55.26 ± 5.3 µM (SEM), which was not statistically different from the literature value, 54.0 ± 12.6 µM (*p* = 0.914) [15]. The mean concentration obtained experimentally for samples from female donors (*n =* 33), 96.4 ± 5.1 µM, was not statistically different from the literature value, 94.8 µM ± 12.9 (*p* = 0.908) [15]. The literature values are represented on the graph by green and blue broken lines for females and males, respectively. The gray dots represent values obtained for individual samples. The red dot represents the mean concentration obtained for each data set. The red horizontal lines represent the upper and lower bounds for the 95% confidence intervals.

The data from the blind study was further analyzed by comparison to known literature values. In this case, each deposit provided was enumerated to best illustrate the spread of data obtained, rather than evaluating data sets by the donor. This information is summarized in Figure 10. The arginine concentration means for males versus female donors in the study were significantly statistically different (*p* < 0.001). For males (*n* = 12), the mean arginine concentration obtained, 55.3 ± 5.3 µM was not statistically different from the literature value, 54.00 ± 12.61 µM (*p* = 0.914) [15]. The mean arginine concentration obtained experimentally for samples from female donors, 96.4 ± 5.1 µM (*n* = 33) was not statistically different from the literature value, 94.80 ± 12.9 µM (*p* = 0.908) [15]. 

## 4. Conclusions

The creation of an optimized microfluidic device to identify biological sex via fingermark deposit analysis is detailed herein. These objectives were achieved by adapting and optimizing the colorimetric Sakaguchi reaction for a microfluidic platform coupled with an inexpensive but objective detection method employing freeware and a standard computer scanner. Jointly, these were used to reliably determine the donor’s biological sex from fingermark content in ≤40 min (following incubation) with mean arginine concentrations that were statistically similar to those previously reported in the literature. The biological sexes of fourteen out of fifteen unknown participants were correctly designated via presumptive testing, demonstrating 93% accuracy of identification using the proposed protocol. Additionally, the compatibility of the proposed method with lifted samples and fingermarks treated with bichromatic magnetic powder was demonstrated. Given the inexpensive and rapid nature of testing methods and required equipment, the potential for reducing assay time, instrument expenditure, and consumption of reagents is evident. In the future, advancements will include developing an integrated analytical instrument with a portable image capture method, on-chip storage of reagents as previously demonstrated by the Landers’ Lab [24,25] and a Peltier system to enable the automation of heating and cooling stages.

## Figures and Tables

**Table 1:** Summary of results obtained from the blind study. The biological sexes of all participants were correctly determined except for one case.

Sample Number	Presumptive ID	Actual ID	Match (Yes/No)
1	Female	Female	Yes
2	Male	Male	Yes
3	Female	Female	Yes
4	Female	Female	Yes
5	Female	Female	Yes
6	Female	Female	Yes
7	Female	Female	Yes
8	Male	Male	Yes
9	Female	Female	Yes
10	Female	Female	Yes
11	Male	Male	Yes
12	Female	Female	Yes
13	Male	Female	No
14	Male	Male	Yes
15	Female	Female	Yes

## Data Availability

The data presented in this study are available on request from the corresponding author. The data are not publicly available due to privacy concerns.
